# Effects of Ultrasound-Assisted Treatment on Physicochemical Properties and Biological Activities of Polysaccharides from *Sargassum*

**DOI:** 10.3390/foods13233941

**Published:** 2024-12-06

**Authors:** Chunxia Zhou, Shanshan He, Shang Gao, Zirui Huang, Wenduo Wang, Pengzhi Hong, Rui-Bo Jia

**Affiliations:** 1 College of Food Science and Technology, Guangdong Ocean University, Guangdong Provincial Key Laboratory of Aquatic Product Processing and Safety, Guangdong Province Engineering Laboratory for Marine Biological Products, Guangdong Provincial Engineering Technology Research Center of Seafood, Guangdong Provincial Engineering Technology Research Center of Prefabricated Seafood Processing and Quality Control, Zhanjiang 524088, China; chunxia.zhou@163.com (C.Z.); 2112203032@stu.gdou.edu.cn (S.H.); gaos1004@163.com (S.G.); wwd@gdou.edu.cn (W.W.); hongpz@gdou.edu.cn (P.H.); 2 School of Agriculture and Biology, Shanghai Jiao Tong University, Shanghai 200240, China; huangzirui@sjtu.edu.cn; 3 Southern Marine Science and Engineering Guangdong Laboratory (Zhanjiang), Zhanjiang 524088, China

**Keywords:** *Sargassum* polysaccharides, ultrasonic treatment, biological activities

## Abstract

The aim of this study was to investigate the effect of ultrasonic treatment on the physicochemical properties and bioactivities of polysaccharides from *Sargassum* samples (SPs) extracted with different solvents. The alkali-assisted extraction of polysaccharide (SPA), acid-assisted extraction of polysaccharides from (SPB), and hot water extraction of polysaccharides (SPCs) were perofrmed on *Sargassum*. Ultrasonic treatment was performed with the SPA, SPB, and SPC in turn, and named USPA, USPB, and UPSC, respectively. The results showed that SPs mainly consisted of mannose, glucose, xylose, rhamnose, galactose, fucose, glucuronic acid, mannuronic acid and guluronic acid. The molecular weight of SPA (434.590 kDa) was the lowest under different solvent extractions, and the molecular weights of SPA, SPB, and SPC were reduced after sonication. SPA had a high carbohydrate content of (52.59 ± 5.16)%, and SPC possessed a high sulfate content of (3.90 ± 0.33)%. After ultrasonic treatment, the biological activities of SPs were significantly increased. The *α*-glucosidase inhibition assay reflected that the IC50 values of the ultrasonic treatment SPs were significantly reduced, and USPA showed the best activity, with an IC50 of (0.058 ± 0.05) mg/mL. Antioxidant assays demonstrated that USPC exhibited greater DPPH- and ABTS-scavenging capacity. In the anti-glycosylation assay, SPs after sonication demonstrated excellent inhibition of glycosylation products and protein oxidation products, with USPA showing the highest inhibition rate. In conclusion, the biological activities of SPs were enhanced after ultrasonic treatment. This study provides a theoretical reference for their use in food and medicines.

## 1. Introduction

Polysaccharides, widely found in plants, animals, and microorganisms, are a class of natural polymers with all kinds of biological functions [1]. In recent years, with the increasing research on polysaccharides, they have proven to have potential bioactive functions, for example, antibacterial, anti-inflammatory, glycemic control, anticancer, antioxidant properties, regulation of intestinal microorganisms [2]. Many polysaccharides with different biological activities are widely used in functional foods and medical research to treat diseases dangerous to human health, such as hypertension, diabetes, and enteritis.

The extraction methods and ultrasonic treatment affect the molecular weight (Mw) distribution, structural characteristics, and biological activities of polysaccharides to different degrees [3]. Currently, the common extraction methods for polysaccharides include solvent extraction, ultrasound-assisted extraction, enzyme-assisted extraction, etc. [4]. It is reported that the biological activity of polysaccharides is closely related to their physicochemical properties (including Mw, monosaccharide composition, sulfate content, etc.) [5]. Extraction methods could modify the different physicochemical properties of polysaccharides [6]. Zheng et al. investigated the different pH values of extraction solutions for the extraction of *Fucus vesiculosus* polysaccharide, and they found that the pH of the extracts had a significant effect on the Mw, apparent structure, and rheological properties, and that polysaccharides extracted at a high pH have better *α*-glucosidase inhibitory activity [7]. Acid-extracted blackberry fruit polysaccharides consisted of lower-Mw and particles, and were found to have better late glycosylation end-product (AGE)-inhibitory activity [8]. Therefore, different extractions have important effects on the physicochemical properties, structure, and activities of polysaccharides.

Appropriate physical treatment has become an effective approach for the preparation of bioactive polysaccharides. Numerous scholars have sought suitable treatment methods to reduce the Mw of polysaccharides to enable them to perform better biological activities. Treatment methods include physical [9], chemical [10], and bio-enzymatic methods [11]. Physical treatment has the merits of reasonable price, environmental friendliness, simplicity of operation, controllable conditions, and high efficiency [12]. Ultrasonic treatment, as a physical method, is greener, more economical, and more environmentally friendly than chemical and enzymatic methods. The principle of ultrasonic treatment is cavitation, which affects the structure of polysaccharides through mechanical bond breaking and cavitation and degrades or modifies polysaccharides [13]. Ultrasonic treatment can depolymerize macromolecular polysaccharides not only by altering the secondary or tertiary structure of the polysaccharides, but also by changing their main and branched chains, increasing their solubility [14]. Chu et al. found that ultrasonic treatment led to a decrease in the Mw of Dendrobium polysaccharides and enhanced their antioxidant activity [15]. Dou et al. found that ultrasonic treatment induced a decrease in the Mw and particle size of blackberry fruit polysaccharides and an increase in their *α*-glucosidase inhibitory activity [8].

*Sargassum* belongs to Phaeophyceae, Fucales, and Sargassaceae [16], and is an edible and economic brown alga that is widely farmed in China and abundant along the coasts of Japan and South Korea. Because it is rich in different kinds chemical components, such as polysaccharides, polyphenols, sterols, and terpenes [17], it is not only used as a food product, but is also regarded as a natural medicine for the treatment of constipation, foot odor, and food accumulation [18]. Polysaccharide is one of the crucial active components of *Sargassum*, accounting for about 40% of its dry weight. Its Mw ranges from about 3.5 kDa to 100 kDa [19]. The proportion of monosaccharides in *Sargassum* varies from region to region and from season to season, but the types of monosaccharides are basically the same. The main components of *Sargassum* polysaccharides are fucoidan and laminarin. Among them, fucoidan is the most important biologically active polysaccharide, which mainly consists of caramel, galactose, mannose, glucose, xylose, and rhamnose [19,20]. The active effects of *Sargassum* polysaccharides are focused on antioxidant [21], anti-inflammatory, immunomodulatory [22], antidiabetic, antitumor, antibacterial and antiviral, and anticoagulant functions [23]. Polysaccharides with a higher Mw are less soluble and might not be favorable to enter an organism through multiple cell membrane barriers to exert pharmacological effects, resulting in lower absorption by the human organism [24]. In addition, Du et al. [25] found that the anti-inflammatory activity of *Sargassum* polysaccharides was enhanced after ultrasonic treatment reduced the Mw of the polysaccharides. The effects of different extraction methods on the physicochemical properties and activities of polysaccharides from *Sargassum* have been reported in the literature. Liu et al. extracted *Sargassum* polysaccharides with hot water, dilute hydrochloric acid, and calcium chloride solution, and compared their physicochemical properties and antioxidant activities [26]. The extraction methods of *Sargassum* polysaccharides are mostly supercritical fluid extraction, pressurized liquid extraction, ultrasound-assisted extraction, enzyme-assisted extraction, and microwave-assisted extraction [19], and the processing methods are relatively simple. Compared with the existing extraction methods, the difference in this experiment is the use of different solvents to extract *Sargassum* polysaccharides, and the ultrasound-assisted treatment of the extracted polysaccharides, compared with the differences in the physicochemical properties of the polysaccharides, as well as their biological activities. In this study, *Sargassum* polysaccharides were obtained under different solvent extraction and ultrasonic treatment conditions. Their chemical composition, structural characteristics, and in vitro activities were compared. In addition, the inhibition of *α*-glucosidase, antioxidant activity, and anti-glycosylation of *Sargassum* polysaccharides were preliminarily investigated. The results of this study provide a reference for further expanding the research fields of polysaccharide activity and functional utilization of *Sargassum.*

## 2. Method and Materials

### 2.1. Material and Reagent

Dried *Sargassum* was obtained from Qingdao Fuxingxiang Import & Export Co., Ltd. (Qingdao, Shandong, China). Monosaccharide standards and *α*-glucosidase were all purchased from Sigma-Aldrich Chemical Co. (St. Louis, MO, USA). 4-Nitrophenyl-*α*-D-glucopyranoside (p-NPG) and 1,1-diphenyl-2-picrylhydrazyl (DPPH) were purchased from Shanghai Aladdin BioChem Technology Co., Ltd. (Shanghai, China). 2,2′-azino-bis-(3-ethyl-benzothiazoline-6-sulfonic acid) (ABTS) was purchased from Shanghai Macklin Biochemical Technology Co., Ltd. (Shanghai, China). All other chemicals and solvents were analytical grade.

### 2.2. Extraction of Sargassum Polysaccharides (SPs)

**Figure 1:**
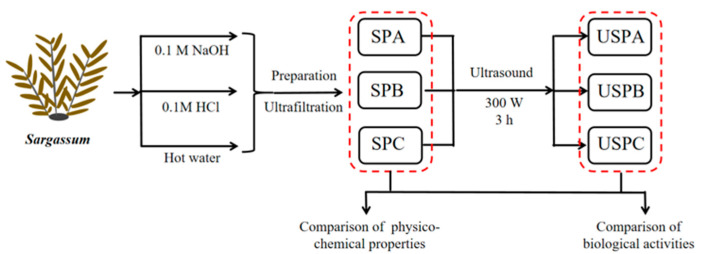
Technology roadmap. (SPA: alkali-assisted extraction of polysaccharide from *Sargassum*; SPB: acid-assisted extraction of polysaccharides from *Sargassum*; SPC: hot water extraction of polysaccharides from *Sargassum*; USPA: ultrasonic treatment SPA; USPB: ultrasonic treatment SPB; and USPC: ultrasonic treatment SPC).

The extraction of polysaccharides from *Sargassum* is shown in Figure 1*. Sargassum* was washed, dried, pulverized, and separated through a 40-mesh sieve. Based on a 1:20 ratio (*w*/*v*), *Sargassum* was mixed with 0.1 M sodium hydroxide solution, 0.1 M hydrochloric acid, and hot water in a blender to form a paste, which was then extracted in a boiling water bath at 90 °C for 2 h. The samples were extracted by filtration through a 200 mesh gauze, the pH was adjusted to neutral (pH = 7), distilled under vacuum, precipitated with four times the volume of anhydrous ethanol for 12 h, centrifuged (8000 rpm, 4 °C, 20 min) in a centrifuge (Thermo Sorvall ST16R, Thermo Inc., Waltham, MA, USA), solubilized, and ultrafiltered five times with ultrafiltration membrane (8 kDa). The three polysaccharide samples (SPA: alkali-assisted extraction of polysaccharide from *Sargassum*, SPB: acid-assisted extraction of polysaccharides from *Sargassum*, SPC: hot water extraction of polysaccharides from *Sargassum*) were lyophilized in a freeze dryer (Alpha 1-4 LD, Christ GmbH, Osterode, Germany) for 48 h. 

### 2.3. Ultrasonic Treatment

Referring to the methods used by others and with minor modifications [27], the 1 mg/mL polysaccharide solutions (SPA, SPB and SPC) were sonicated at 300 W for 3 h at room temperature using an ultrasonic cleaner (KQ-300DE, Kunshan Ultrasonic Instrument Co., Ltd., Kunshan, China). The temperature of the medium was 50 ± 3 °C after 3 h of ultrasonic treatment. Three ultrasonic-treated polysaccharides were obtained via lyophilization (USPA: ultrasonic treatment SPA; USPB: ultrasonic treatment SPB; and USPC: ultrasonic treatment SPC).

### 2.4. Chemical Composition Analysis

Carbohydrates were determined using *L*-fucose as the standard; the total phenol content was measured using the forintol method with gallic acid as the standard [28]. Sulfate content was analyzed using the barium sulfate turbidimetric method, with potassium sulfate as the standard [29]. The protein content was determined using the Bradford assay method [28].

The monosaccharide composition was measured using a Waters e2695 high-performance liquid chromatography system (Waters Corporation, MA, USA) equipped with ultraviolet detection. In short, the SPs sample (10 mg) was hydrolyzed with trifluoroacetic acid (4 M) at 110 °C for 8 h. Methanol was added to remove excess trifluoroacetic acid. The hydrolysate was obtained by reacting with 1-pheny l-3-methy l-5-pyrazolinone (PMP) for 100 min at 70 °C. Finally, the sugar derivatives were filtered onto a C18 chromatographic column.

### 2.5. Determination of Mw

The samples (1 mg/mL) were dissolved in 0.1 M NaNO_3_ aqueous solution (containing 0.02% NaN_3_, *w*/*w*), filtered through a filter with a pore size of 0.45 μm, and then tested on a gel chromatography–differential exponential-multi-angle laser light scattering system.

### 2.6. Measurement of Particle Size and Zeta Potential

The SPs samples were diluted with deionized water (1 mg/mL), and the particle size and zeta potential were evaluated using a laser particle sizer (NanoBrook Omni, Bruker, New York, USA).

### 2.7. Fourier Transform Infrared Spectrometry (FT-IR) Analysis

The SPs samples and KBr powders were mixed and pressed into tablets. Spectra were recorded on a Bruker VERTEX 33 spectrometer (Bruker, Karlsruhe, Germany) in the range of 4000 to 400 cm^−1^.

### 2.8. Water-Holding Capacity (WHC)

The WHC (g/g) of the samples was tested with reference to the previous method, with slight modification [6]. In detail, 0.02 g of sample (M_0_) and 10 mL of centrifuge tube (M_1_) were accurately weighed; then, the accurately weighed sample was added and 50 mL of deionized water was added to the pre-weighed centrifuge tube of the previous step, placed it in freezer, and then centrifuged (12,000 rpm, 4 °C, 30 min). The supernatant was discarded, and the total weight (M_2_) of the centrifuge tube was weighed whilst containing the sample after water absorption. The formula for WHC is as follows (1):

(1) $$WHC\left(g/g\right)=\frac{\left(M_{2}-M_{1}-M_{0}\right)}{M_{0}}$$

### 2.9. Oil-Binding Capacity (OBC)

Referring to Zhang et al. [30], 0.5 g of sample (M_0_) and 10 mL centrifuge tube (M_1_) were weighed accurately. Then, the SPs samples weighed in the previous step were added to the centrifuge tube weighed in the previous step, along with 5 mL of peanut oil. The samples were allowed to stand at 25 °C for 10 h and then centrifuged (12,000 rpm, 4 °C, 30 min). The supernatant oil phase was removed, and the total weight (M_2_) of the centrifuge tube containing the adsorbed peanut oil sample was weighed. The formula for calculating the OBC is as follows (2):

(2) $$OBC\left(g/g\right)=\frac{\left(M_{2}-M_{1}-M_{0}\right)}{M_{0}}$$

### 2.10. Rheological Property

The viscosity of SPs (20 mg/mL) was analyzed using a rheometer (HAAKE MARS, Thermo Electron, Waltham, MA, USA) equipped with a C60 parallel plate at 25 °C with a shear rate of 1–100 s^−1^.

### 2.11. Scanning Electron Microscopy (SEM)

A certain amount of SPs powder was used and fully dried. The samples were fixed on the sample stage with conductive glue. The morphology of the SPs were observed using a scanning electron microscope JSM-7500F (JEOL, Tokyo, Japan) after gold spraying.

### 2.12. α-Glucosidase Inhibition Assay

Following the practice of previous studies with minor modifications [31], different concentrations of SPs were mixed with *α*-glucosidase, and 4-Nitrophenyl-*α*-D-glucopyranoside (p-NPG) substrate was added and incubated for 20 min at 37 °C. Finally, the termination solution (Na_2_CO_3_) was added. The absorbance was recorded at 405 nm and the formula is as follows (3):

(3) $$Inhibiton\left(\%\right)=\left[1-\frac{\left(A_{test}-A_{test\;blank}\right)}{\left(A_{control}-A_{control\;blank}\right)}\right]\times 100\%$$

where A_test_ is the light absorption value of the sample, *α*-glucosidase, and p-NPG reaction solution; A_test blank_ is the light absorption value of the sample solution; A_control_ is the light absorption value of the *α*-glucosidase and p-NPG reaction solution; and A_control blank_ is the light absorption value of the buffer solution.

### 2.13. Antioxidant Activity

The antioxidant assay was used with reference to the work by Bu et al. [32]. DPPH scavenging activity: A 0.2 mM DPPH stock solution was prepared and protected it from the dark for 24 h for later use. Then, 2 mL of the sample solution and 2 mL of DPPH solution were mixed and protected from light for 30 min. The absorbance value of A_1_ was recorded at 517 nm, the absorbance value of 2 mL of ultrapure water and 2 mL of DPPH was recorded as A_2_, and the absorbance value of 2 mL sample and 2 mL of absolute ethanol was recorded as A_3_. The scavenging capacity of the samples for DPPH were calculated using the following Formula (4), and the final results were expressed in Trolox equivalents.

(4) $$DPPH\;radical\;scavening\;\left(\%\right)=\frac{1-\left(A_{1}-A_{3}\right)}{A_{2}}\times 100\%$$

ABTS scavenging activity: ABTS mother liquor was prepared with potassium persulfate and kept in the dark. The ABTS mix was diluted with PBS buffer solution (pH = 6.8) and the absorbance value at 734 nm was 0.70 ± 0.02. The sample was then mixed with the ABTS diluent, protected from light, and recorded as A_1_. PBS and a series of different concentrations of Trolox were used as blank and positive controls, and their absorbance values were denoted as A_2_ and A_3_, respectively. The formula was expressed as follows (5):

(5) $$ABTS\;radical\;scavening\;\left(\%\right)=\frac{\left(A_{2}\;-A_{1}\;\right)}{A_{3}}\times 100\%$$

The standard curve regression equation was used according to the Trolox concentration and ABTS free radical scavenging rate. The TEAC (Trolox-equivalent antioxidant capacity) value of the sample was calculated from the equation and clearance, and the results were expressed in mM TE/g.

### 2.14. Anti-Glycation Activity

According to a previous study [33], the glycosylation of bovine serum albumin–glucose was modeled using aminoguanidine (AG) as a positive control. Then, the inhibition rates of the SPs against glycosylation products (fructosamine, dicarbonyl compounds, and AGEs) and protein oxidation products (dityrosine, kynurenine, and *N’*-formylkynurenine) were tested separately using a fluorescence spectrophotometer (Luminous, Thermo Electron, Waltham, MA, USA).

### 2.15. Statistical Analysis

All data were statistically analyzed using SPSS (version 23.0) software. All results were expressed as mean ± standard (*n* = 3) deviation. Significant differences (*p* < 0.05) were considered statistically significant.

## 3. Results and Discussion

### 3.1. The Chemical Composition of SPs

The carbohydrate, total phenol, protein, sulfate content, Mw, and monosaccharide composition of the SPs are shown in Table 1. Different extraction solvents had a significant impact on the chemical composition of polysaccharides. The carbohydrates of the SPs were in the range of 40.23%~52.59%, and SPA had the highest carbohydrates among them. The contents of total phenol and proteins were 0.62%~1.03% and 0.33%~1.89%, respectively. The sulfate content was in the range of 2.89%~4.86% (*p* < 0.05). The results pointed out that the different extraction solvents could affect the chemical composition of the SPs.

The SPs samples were mainly composed of fucose (23.27%~36.77%), galactose (12.97%~16.91%), glucose (0.98%~2.00%), xylose (5.48%~7.92%), mannose (7.31%~9.55%), guuronic acid (3.66%~3.85%), and glucuronic acid (11.12%~15.18%). However, SPB and SPC contained rhamnose (1.10%~1.61%), and SPA contained a large amount of mannoric acid (11.97%~35.01%) (Table 1). The results showed that the monosaccharide composition and proportion of the SPs extracted from different solvents varied slightly.

### 3.2. The Mw of SPs

The Mw of the SPs was 434.590~1073.294 kDa (Table 1). Compared with SPC, SPA and SPB had a lower Mw (*p* < 0.05), which is in line with the reported conclusion that acid and alkali extraction can destroy the glycosidic bonds of the polysaccharides, resulting in a decrease in Mw [34]. After ultrasonic treatment, the Mw of SPA, SPB, and SPC were reduced to USPA (375.354 kDa), USPB (718.293 kDa), and USPC (895.089 kDa), respectively. Jia et al. performed a treatment (65 W, 1 h) of corn silk polysaccharide with ultrasound, which resulted in a decrease in the Mw of the polysaccharide from 7.23 × 104 Da to 5.49 × 104 Da [35]. Therefore, different solvent extracts severely affected the Mw of SPs, which decreased after ultrasonic treatment.

### 3.3. Zeta Potential and Particle Size Distribution of SPs

**Figure 2:**
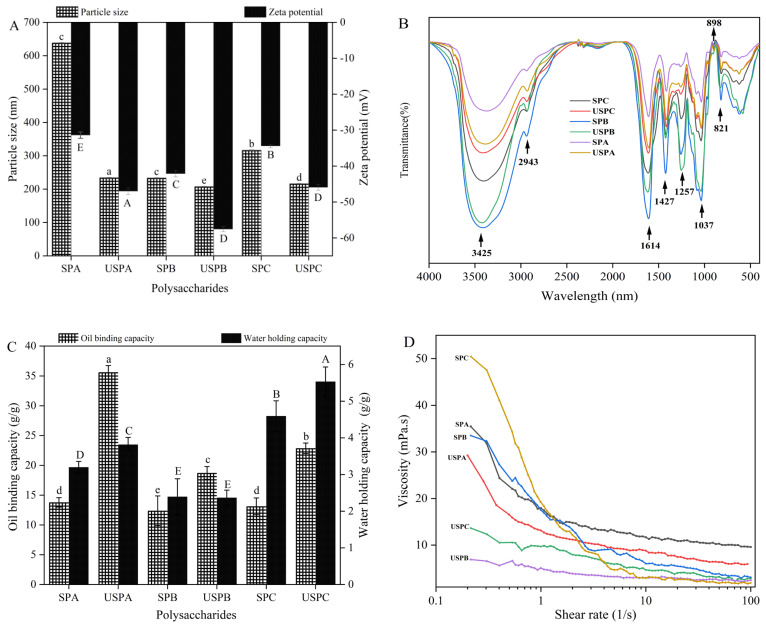
Particle size and zeta potential (**A**); FT-IR (**B**); oil-binding capacity (OBC) and water-holding capacity (WHC) (**C**); and viscosity (**D**) of SPs. Data are expressed as mean ± SD (*n* = 3). Significant differences (*p* < 0.05) are indicated with different letters.

Zeta potential is an important indicator used to characterize the stability of a dispersed system of polysaccharide solutions. The higher the absolute value of the zeta potential, the better stability of the solution [36]. In Figure 2A, the SPs are negatively charged, indicating that the SP solutions stabilized well, which could be attributed to the abundant electrostatic repulsion in their zeta potentials, with all being greater than 30 mV in absolute value [37]. According to previous reports, the absolute value of zeta potential is related to the content of carbohydrates, aldoses, and sulfate ions [38]. These results show that the higher absolute values of zeta potential of alkali-extracted and acid-extracted polysaccharides were related to the higher number of carbohydrates. The absolute values of the zeta potentials of the SPs after sonication were all higher than those before no sonication, indicating that sonication can enhance the stability of polysaccharide solutions, which is consistent with the phenomenon found in previous studies [39]. The particle sizes were 538.47 nm (SPA), 233.49 nm (SPB), and 316.7 nm (SPC). The larger particle sizes of SPA (or SPC) compared to SPB might be due to the increased level of uric acid cross-linking leading to polysaccharide aggregation. Correspondingly, the particle size of SPs decreased significantly (*p* < 0.05) after degradation via ultrasound, indicating that ultrasound treatment was able to reduce the particle size of polysaccharides [40], which is in agreement with the scanning electron microscopy results.

### 3.4. FT-IR Spectroscopy of SPs

Fourier-transform infrared spectroscopy was used to analyze the characteristic functional groups of SPs. In Figure 2B, the strong and broad absorption peak at 3425 cm^−1^ was caused by the stretching vibration of O-H [41]. The absorption peaks at 2943 cm^−1^ and 1427 cm^−1^ were caused by the stretching vibration and bending vibration of C-H, respectively [42]. The absorption peak at 1614 cm^−1^ was caused by the stretching vibration of C=O, which indicates that SPs contained glyoxalic acid [43]. The absorption peak at 1257 cm^−1^ was caused by the asymmetric stretching vibration of O=S=O, which proves the existence of a sulfuric acid group [44]. Furthermore, 1037 cm^−1^ is the characteristic absorption peak of the pyran ring, and 898 cm^−1^ is the characteristic absorption peak of the *β*-glycosidic bond. Moreover, 821 cm^−1^ is the absorption peak caused by the stretching vibration of the *α*-glycosidic bond [45]. Comparing the infrared spectra of the six polysaccharides, there was no obvious displacement of the characteristic absorption peaks, indicating that the various extraction methods and ultrasonic treatment did not lead to changes in the functional groups of SPs.

### 3.5. WHC and OBC of SPs

WHC is one of the important indexes that can be used to show hydration properties. As shown in Figure 2C, there was a significant difference in the WHC of SPs, and after ultrasonic treatment, the WHC and solubility of SPs were significantly increased (*p* < 0.05) [46], which might be related to the particle size, Mw, and inter-chain association. SPA had the highest WHC value, which might depend on the low Mw and particle size, leading to the exposure of more hydrophilic group sites, thus increasing the hydration of the particles [47]. Ultrasonication significantly (*p* < 0.05) enhanced the WHC solubility of SPs. After ultrasound treatment, the glycosidic bonds of polysaccharides were also randomly broken and the particle size was reduced to form a loose and porous structure, which also affects the WHC of the sample. This result is consistent with a previous study [48].

OBC is a prominent functional property. In Figure 2C, the OBC values of SPA, SPB, and SPC in this experiment are basically comparable at 13.78 g/g, 12.37 g/g, and 13.12 g/g, respectively. However, after ultrasound treatment, the OBC value of USPA increased to 35.62 g/g (an increase of 61.31%), and those of USPB and USPC also significantly increased by 46.93% and 40.44%, respectively (*p* < 0.05). The results prove that the SPs had a certain OBC. And the OBC values increased significantly after physical treatment, which might be attributed to the reduced particle size, increased specific surface area, and loose porous structure after ultrasonic treatment. Similar conclusions are reached by He et al. [49].

### 3.6. Apparent Viscosity Analysis of SPs

Polysaccharides are used as thickeners, gelling agents, and emulsifiers in the food industry because of their good rheological properties [50]. As shown in Figure 2D, the highest viscosity of SPC might be attributed to the high Mw, which led to the easy entanglement of the polysaccharide chains. SPA and SPB were the next most viscous, which might be attributed to their structures with a high content of glycoalkalic acid. The apparent viscosity of physically degraded SPs was negatively correlated with the shear rate, indicating that SPs were pseudoplastic fluids. With the increase in the shear rate, the entanglement of the polysaccharide chains was weakened. The dispersed polysaccharide chains were gradually arranged in an orderly manner according to shear, and the interaction between polysaccharide chains was weakened, resulting in a decrease in apparent viscosity [51]. The intermolecular interactions of high Mw polysaccharides were stronger, resulting in the easy entanglement of polysaccharide chains. After ultrasonic treatment, the apparent viscosity of SPs decreased, which is consistent with the study of Wu et al. [52].

### 3.7. SEM Analysis of SPs

**Figure 3:**
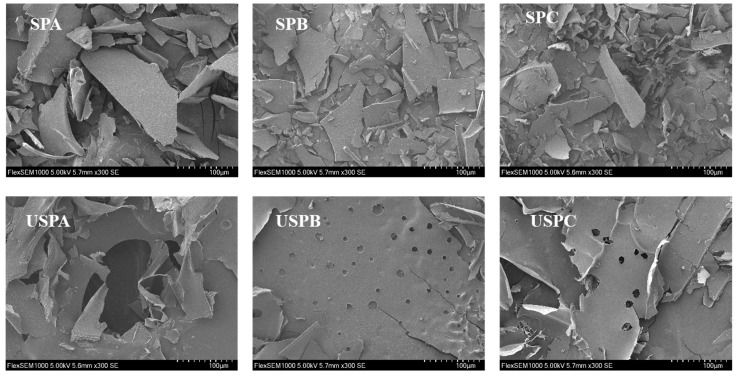
SEM images of SPs (magnification: 300×).

SEM is an effective tool for observing the microscopic surface morphology of polysaccharides. The images of SPs are shown in Figure 3. The surfaces of SPA, SPB, and SPC were smooth, dense, and irregularly lamellar. The SEM image of SPA showed large and irregular fragments with a smooth surface, which might be due to the aggregation of total sugars, consistent with the results of particle size determination. The surface of SPB was slightly rough with many fine lumpy particles, which might be due to the aggregation of glucuronic acid cross-links. SPC showed relative roughness, with a fine fragmented structure. After ultrasonication, the SPs showed significant differences in morphology and shape. The SEM images of USPA, USPB, and USPC showed a fragmented appearance and porous structure, which might be attributed to the accelerated diffusion and dissolution of polysaccharides by transient high pressure, turbulent shear, and strong physical cavitation effects [53].

### 3.8. Analysis of α-Glucosidase Inhibition

**Figure 4:**
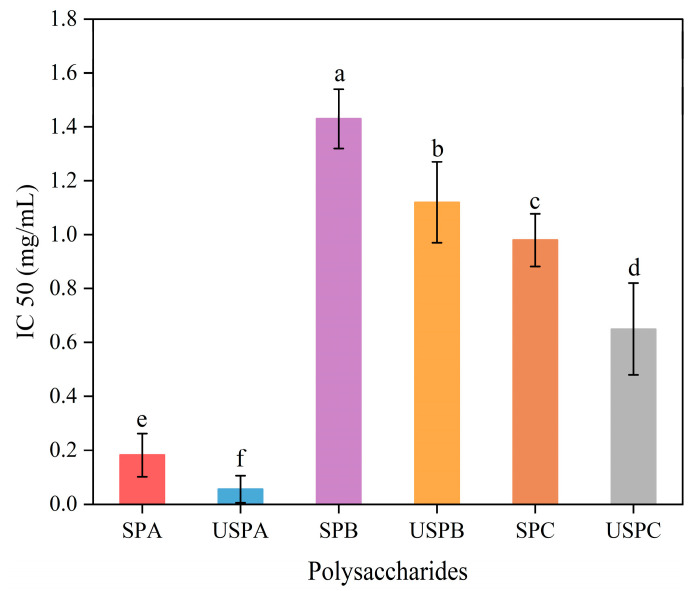
*α*-glucosidase inhibition assay of SPs. Data are expressed as mean ± SD (*n* = 3). Significant differences (*p* < 0.05) are indicated with different letters.

The inhibition of *α*-glucosidase activity is one of the most effective methods used to control hyperglycemia. In this experiment, the potential ability of SPs to lower blood glucose was initially evaluated by using an *α*-glucosidase inhibition assay. All the SPs demonstrated concentration-dependent inhibition of *α*-glucosidase. As shown in Figure 4, the IC50 values were 0.182 ± 0.08 mg/mL (SPA), 0.98 ± 0.098 mg/mL (SPC), and 1.43 ± 0.11 mg/mL (SPB) in descending order (*p* < 0.05). The reason behind the strong inhibitory effect shown by SPA might be related to its low Mw and high glucuronic acid content. Fu et al. [54] reported that the ability of the -COOH group on the sugar chain of loquat leaf polysaccharides to form strong acting hydrogen bonds with residues on *α*-glucosidase resulted in a decrease in the activity of the glycosidase. After ultrasonic treatment, the IC50 of the SPs extracted with different solvents was reduced, with USPA having the lowest IC50 value (0.056 ± 0.05 mg/mL), 69% lower compared to the SPA; the IC50 was 21.6% lower in the USPB than the SPB; the USPC IC50 was 33.7% lower than SPC. The ultrasonication brought the low-Mw polysaccharides closer to the active site of *α*-glucosidase, causing a conformational change in the enzyme, thus leading to inactivity. The same results were found by Guo et al. [55]. The results demonstrate that different solvent extractions affected the *α*-glucosidase activity, and a certain degree of physical treatment increased the *α*-glucosidase inhibitory activity. The high *α*-glucosidase inhibitory activity of polysaccharides might be due to the lower Mw and greater number active sites exposed. Xiong et al. also found that alkali-extracted *Evodiae fructus* polysaccharides had stronger *α*-glucosidase inhibitory activity than acid-extracted *Evodiae fructus* [56].

### 3.9. Antioxidant Capacity Analysis

**Figure 5:**
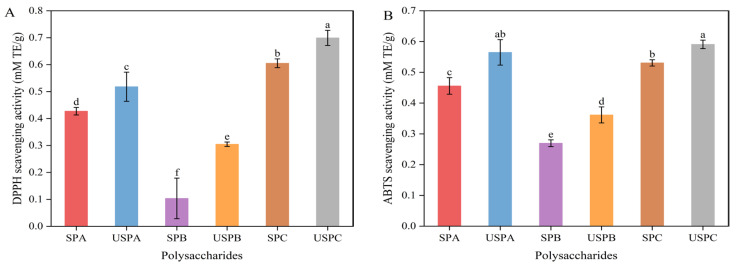
Antioxidant test, including DPPH (**A**) and ABTS (**B**) free radical scavenging capacity. Data are expressed as mean ± SD (*n* = 3). Significant differences (*p* < 0.05) are indicated with different letters.

As shown in Figure 5A,B, the SPs showed significant scavenging ability against DPPH and ABTS free radicals (*p* < 0.05). Compared with SPB, SPA and SPC had better free radical scavenging capacities for DPPH (0.427 ± 0.014, 0.605 ± 0.016 mM TE/g) and ABTS (0.456 ± 0.027, 0.531 ± 0.102; mM TE/g), respectively. The higher-Mw SPC had better free radical-scavenging capacity than SPA and SPB, which was similar to the results by Chen et al. [34]. DPPH and ABTS radical scavenging were significantly enhanced in ultrasound-degraded SPs (*p* < 0.05), with a consistent trend. The inhibition of DPPH and ABTS radicals was increased by SPA (21.25% and 23.9%), SPB (65.97% and 34.13%), and SPC (15.58% and 11.3%), respectively. The results indicate that ultrasonic treatment enhanced the antioxidant activity of SPs, which might be related to the decrease in Mw, particle size, and viscosity, as well as the increase in glyoxylate content induced by ultrasonic treatment. Yan et al. [57] found that the antioxidant activity was enhanced with the decrease in the Mw of *Phellinus linteus* mycelia polysaccharides caused by ultrasonic treatment.

### 3.10. Anti-Glycosylation Assay

Long-term chronic hyperglycemia can induce the formation of AGEs, and AGEs are closely associated with diabetic complications such as vascular sclerosis, nephropathy, and atherosclerosis [3]. Fructosamine, dicarbonyl compounds, and AGEs are the products of three periods of glycosylation activity [58]. The inhibition of the production of each period’s products could reduce the end-product AGEs. Non-enzymatic glycosylation reactions are usually accompanied by oxidative activities, and the tryptophan and tyrosine residues of proteins are easily damaged by sugar oxidation; inhibiting the production of protein oxidation products can protect proteins from oxidation [59].

**Figure 6:**
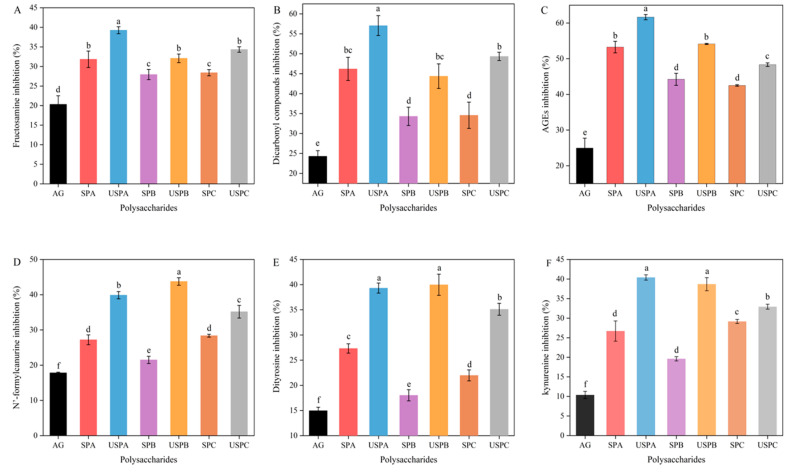
Anti-glycosylation assay, including the inhibition of fructosamine (**A**), dicarbonyl compounds (**B**), and AGEs (**C**), di-tyrosine (**D**), kynurenine (**E**), and *N’*-formyl kynurenine (**F**). Data are expressed as mean ± SD (*n* = 3). Significant differences (*p* < 0.05) are indicated with different letters.

As shown in Figure 6A–F, the SPs expressed good inhibition (*p* < 0.05) of the products of all three stages of glycosylation, with the effects all being better than that of the positive control, aminoguanidine (AG). Among the SPs, SPA demonstrated the strongest inhibitory effects on fructosamine, dicarbonyl compounds, and AGEs, which could be attributed to the lower Mw and higher content of glucuronic acid content. This is the same as the results reported by Gong et al. [3]. The inhibition rate of SPs against the glycosylation assay is shown in Figure 6A–C. Compared with SPB, SPA and SPC had better inhibition rates against glycosylation products (*p* < 0.05). In the primary stage, SPA and SPC inhibited fructosamine products at the rate of 31.84% and 28.40%, respectively. In the intermediate stage, SPA and SPC inhibited *α*-dicarbonyl compounds at the rate of 46.21% and 34.58%, respectively. And in the final stage, *α*-dicarbonyl compounds induced the rapid cross-linking of proteins to produce stable end product AGEs, and The inhibition rates of the AGEs were 53.25% and 42.96%, respectively. The inhibitory effects of the SPs after ultrasound treatment were stronger than those of untreated polysaccharides (*p* < 0.05). This indicates that the SPs had better anti-glycosylation effects after ultrasound treatment because after ultrasound treatment, the Mw of the SPs was reduced, the particle size became smaller, and the specific surface area became bigger. This resulted in more sites to bind with free radicals, both shielded or terminated radicals, and effectively inhibited the oxidative reactions triggered by the free radicals, thus blocking the non-enzymatic glycosylation reaction at the oxidative stage [60]. The inhibition rate of USPA reached more than 50% at the middle and end stages, indicating that the inhibition mainly occurred at this stage.

Upon the destruction of proteins via sugar oxidation, tryptophan residues were modified to form di-tyrosine and *N’*-formyl kynurenine. In contrast, tyrosine residues were modified to form kynurenine, and the different characteristic fluorescence peaks of these products of sugar oxidation were regarded as markers of protein oxidation [58]. As shown in Figure 6D–F, the SPs showed a strong inhibition of the formation of di-tyrosine, kynurenine, and *N’*-formyl kynurenine, and the ultrasonically degraded SPs exhibited better inhibition, all of which were superior to the positive AG (*p* < 0.05). Common antioxidants, such as catechin, gallate, quercetin, and sanguinarine, have been reported to inhibit the glycation reaction [61]. SPA and SPC were more effective than SPB, probably due to the fact that SPA and SPC had a better antioxidant capacity, which is in line with the findings of a previous report [43]. The SPs could better inhibit the generation of protein oxidation products after sonication, suggesting that a certain degree of sonication treatment can enhance the anti-glycosylation effect of SPs and protect proteins from oxidation.

## 4. Conclusions

In this study, different solvents were used to extract polysaccharides from Sargassum, and the polysaccharides were sonicated. The results reveal that the different solvents had an impact on the Mw, monosaccharide composition and ratio, and physicochemical properties of the SPs, but ultrasonic treatment had little effect on the chemical composition. Ultrasonic treatment decreased the Mw and particle size of the polysaccharides from *Sargassum* and increased their oil- and water-holding capacity. Meanwhile, sonication generated greater biological activities. Among the polysaccharides, USPA demonstrated the strongest inhibitory activity against *α*-glucosidase (IC50 = 0.056 ± 0.05 mg/mL). Furthermore, USPA and USPC had superior antioxidant capacity, and USPA exhibited better anti-glycosylation ability and protected the protein from oxidation. Overall, this study lays the foundation for the use of *Sargassum* polysaccharides in functional foods and drugs.

## Figures and Tables

**Table 1:** Chemical composition and average Mw of SPs.

Items	SPA	USPA	SPB	USPB	SPC	USPC
Carbohydrate (%)	52.59 ± 5.16 ^a^	49.12 ± 3.52 ^b^	50.26 ± 1.52 ^b^	45.08 ± 3.08 ^c^	40.23 ± 3.59 ^d^	41.65 ± 1.31 ^d^
Total phenol (%)	0.81 ± 0.7 ^c^	0.88 ± 0.6 ^b^	0.62 ± 0.3 ^d^	0.68 ± 0.2 ^d^	0.99 ± 0.5 ^a^	1.03 ± 0.6 ^a^
Protein (%)	1.72 ± 0.14 ^b^	1.89 ± 0.03 ^a^	1.01 ± 0.01 ^d^	1.15 ± 0.20 ^c^	0.33 ± 0.10 ^f^	0.47 ± 0.12 ^e^
Sulfate (%)	3.76 ± 0.63 ^c^	4.58 ± 0.52 ^b^	3.90 ± 0.33 ^c^	4.86 ± 0.41 ^a^	2.89 ± 0.34 ^e^	3.66 ± 0.45 ^d^
Monosacchride composition (%)						
Mannose	7.62	7.31	9.55	8.99	8.67	8.99
Glucose	1.13	0.98	1.80	1.75	2.00	1.99
Rhamnose	-	-	1.61	1.40	1.10	1.13
Galactose	12.97	12.28	17.42	16.64	16.70	16.91
Xylose	5.51	5.48	7.92	7.69	6.50	6.67
Fucose	23.27	24.15	36.77	36.46	26.86	27.01
Glucuronic Acid	11.42	11.12	12.96	15.18	13.32	12.95
Mannuronic Acid	34.22	35.01	11.97	14.90	24.85	24.36
Guluronic Acid	3.85	3.66	-	-	-	-
Molecular weight (kDa)						
Mw	434.590	375.354	786.198	718.293	1073.294	895.089
Mn	161.231	121.688	82.651	69.941	287.163	211.544
Mw/Mn	2.695	3.085	9.512	10.270	3.738	4.231

Data are expressed as mean ± SD (*n* = 3). Significant differences (*p* < 0.05) are indicated with different letters.

## Data Availability

The data presented in this study are available on request from the corresponding author. The data are not publicly available due to privacy restrictions.
